# Clinical outcome of right ventricle outflow tract management for repair of Tetralogy of Fallot with three contemporary surgical strategies

**DOI:** 10.12669/pjms.37.5.3961

**Published:** 2021

**Authors:** Tariq Waqar, M. Zubair Ahmed Ansari, Kamran Khan

**Affiliations:** 1 Tariq Waqar, (FCPS, FRCS), Associate Professor, Pediatric Cardiac Surgery, Punjab Institute of Cardiology, Lahore, Pakistan; 2 M. Zubair Ahmed Ansari, (FCPS), Assistant Professor, Cardiac Surgery, Cardiac Centre, Bahawalpur, QMC, Bahawalpur, Pakistan; 3 Kamran Khan, (MS), Medical Officer, Punjab Institute of Cardiology, Lahore, Pakistan

**Keywords:** Mono-cusp repair, Pulmonary valve repair, Tetralogy of Fallot, Trans-annular patch

## Abstract

**Objective:**

To compare the early operative outcome of TOF repair with three contemporary repair strategies of RVOTO repair i.e. TAP, Mono-cusp construction (MC) in TAP and pulmonary valve repair.

**Methods:**

Study is performed at Punjab Institute of Cardiology, Lahore from May 2016 to April 2020. Retrospective analysis of data was performed for patient who underwent TOF repair by three different strategies of RVOT repairs during TOF surgery based on z scoring for pulmonary valve annulus. Group-I underwent trans-annular patch repair, while Group-II and III underwent Mono-cusp repair with autologous pericardium and pulmonary valve repair respectively. Analysis of Variance (ANOVA) and Pearson Chi-Square (PCS) statistics were used to compare the three groups for numeric and categorical variables respectively. Post-hoc t-test and Bonferroni correction were performed for numeric data to compare two groups with each other. Chi-square test was used to perform comparison between groups for categorial variables.

**Results:**

ANOVA for aortic cross clamp time, total CPB time, Post-operative mechanical ventilation time, ICU stay and hospital stay showed statistical difference among all three group with p-value less than 0.05 however post hoc T-test showed this variation is limited to post-operative mechanical ventilation only when groups compared with each other. PCS showed there was difference for incidence of difficult weaning from CPB when all three groups compared while there was no difference in operative mortality with p-value of 0.15. However, Group-II comparison with Group-I showed that weaning from CPB was superior in-Group-II with p-value of 0.016. Group-III showed the best statistics for all operative outcome variables among all three groups. Comparison of incidence of post-operative moderate pulmonary regurgitation before discharge between Group-II and Group-III showed significant difference with p-value of 0.0052.

**Conclusion:**

PV repair strategy should be employed for RVOT repair of TOF whenever feasible. MC repair showed fewer hours of postoperative mechanical ventilation and higher incidence of easy weaning from CPB when compared to TAP, however its impacts over ICU stay, Hospital stay and operative mortality is not profound in our TOF repair population.

## INTRODUCTION

Tetralogy of Fallot (TOF) is characterized by ventricular septal defect (VSD), right ventricular outflow tract (RVOT) obstruction, Right ventricle (RV) have diversities hypertrophy and over-riding of aorta.^1^ Clinically these components of TOF in three-dimensional orientation along with variation in severities of narrowing of RVOT and sizing of VSD. In addition, many other associated congenital malformations can also be coexisting like patent ductus arteriosus (PDA), major aorto-pulmonary collaterals (MAPCAs), absent pulmonary valve small pulmonary artery and it branches etc.^2^ Thus, every TOF presents somewhat differently during surgical handling and manipulation. Surgical repair of TOF is mainly based on closure of VSD and relieving of RVOT obstruction with ultimate improvement in all four components of congenital anomaly. Thus, success of surgical repair lies in optimal management of two above-mentioned parts of procedure. RVOTO of TOF is caused at different levels i.e. 10% have PV stenosis, 50% have infundibular stenosis, 30% have mixture of above two while remaining have other more complex presentation like atresia of PV or pulmonary artery stenosis etc.^3^

Severity of TOF is directly related to severity of PV annular hypoplasia. z-scoring is utilized as objective and scientific method to demonstrate the severity of PV annular hypoplasia in surgical practice. Classically, Z score of -2 or less signifies that annular preservation may result in significant residual RVOTO and thus mandate annuloplasty to enlarge RVOT for successful repair. Conversely, Z score of more than -2 favors strategy for PV preservation.^4^ TAP is characterized by inherent free pulmonary regurgitation while trivial to mild in case of PV sparing TOF repair. Both trans-annular patch (TAP) and PV preservation have shown promising result in corrective surgery for TOF. However, much evidences have demonstrated superior short term and long-term operative results of PV preservation.^5^,^6^ Therefore many of expert congenital cardiac surgery groups started to employ repair strategies that halt free pulmonary insufficiency to improve operative results. Mono-cusp repair in TAP is one of these. Creation of mono-cups in TAP and hence less incidence of free PR is theoretically expected to yield favorable impact over right ventricle function and clinical outcome.^7^

The objective of our study was to compare the early outcome of TOF repair with three contemporary repair strategies of RVOTO repair i.e. TAP, Mono-cusp construction in TAP and pulmonary valve preservation. The purpose is to define the beneficial impact of mono-cusp construction by comparing it to other two repair options.

## METHODS

This study was conducted at Punjab Institute of Cardiology, Lahore. Study approval was taken from ethical committee and institute review board (Ref No.: RTPGME-Research-129, dated June 2, 2020). Data was collected retrospectively from May 2016 to April 2020 for patients who underwent surgery for TOF repair. The patient file, operative theater record, intensive care record, preoperative echocardiography and postoperative echocardiography record were utilized to retrieve study related data. During our congenital cardiac surgery practice for above mentioned period of time, every patient of TOF underwent pre-operative echocardiography and cardiac catheterization as pre-operative work-up to clarify the anatomy of TOF and associated cardiac anomalies. We performed the Z score of pulmonary valve annulus for our patient population with online calculator based on Petersen et al study.^8^ Their study population ranged from 1-18 years. The calculator needs age (years), weight (kg), height (cm), Body surface area (BSA- cm2) and pulmonary valve area (mm) measured by echocardiography. This calculator yield Z score as - Z score, 0 or + z-score values and normal range of PV annulus as millimeter. We used Z score of -2 or below as guide for TAP or mono-cusp construction for RVOTO repair in our TOF population. Patient with Z score of above -2 were offered pulmonary valve repair. A total of 175 patients were operated for TOF repair. Standard cardiopulmonary bypass technique with cold blood antegrade cardioplegic arrest strategy was used for procedures in all case. Cases were segregated into three groups on the basis of repair strategy for RVOTO i.e. Trans-annular patch repair (Group-I), Mono-cusp repair (Group-II), and pulmonary valve repair (Group-III). Trans-annular repair is characterized by vertically slit in the ROVOT across the pulmonary annulus from PA to RV infundibulum and closing the defect with autologous pericardial patch with resultant relieve in RVOTO and free pulmonary regurgitation (PR). Monocusp reconstruction repair performed by using autologous pericardium that was treated with Glutaraldehyde for 20 seconds. RVOT is opened across the pulmonary annulus, native pulmonary valve leaflet is left intact, opened RVOT is repaired with trans-annular pericardial patch with pericardium made mono-cusp sewn from the one edge of RVOT across the trans-annular patch to the other edge of RVOT (Attached margin of Mono-cusp valve). The free mobile edge of constructed mono-cusp valve lies above the native pulmonary valves leaflet. Pulmonary valve repair is characterized by performing pulmonary commissurotomy to enrage the effective orifice area of pulmonary valve and preserve its competency.

Age (years), weight (kg), gender (male, female), aortic cross clamp time (mins), total cardiopulmonary bypass time (CPB, mins), difficulty in weaning from CPB, post-operative ventilation time (hours), ICU stay (hours), hospital stay (Days) were retrieved from hospital record. Difficult weaning from CPB was defined as either need for higher inotropic support (0.1 microgram/kg/min of adrenaline or 0.20 microgram/kg/min dobutamine) or more than 30 mins of empty heart beating on CPB while initial attempt of liberation from CPB resulted in cardiac dilatation or combination of both. Operative notes were use to get fine operative details including RVOT repair strategy to segregate patient into different categories. Postoperative moderate pulmonary regurgitation was noted for Group-II and Group-III from pre-discharge trans-thoracic echocardiography. Microsoft excel 2016 was utilized to analyze the data. Frequency was measured for categorical data like gender, difficult weaning from CPB, and operative mortality. Mean and standards deviation of mean was measured for numeric data like age, cross clamp time, CPB time, Ventilation time, ICU stay and hospital stay etc. for all three groups. Single factor analysis of variance (ANOVA-test) was performed to find statistical significance (p-value) between the groups. P-value of 0.05 or less was specified as significant. Then we performed the post-hoc t-test that compares the statistical difference between two groups (Group-I vs Group-II, Group-I vs Group-III and Group-II vs Group-III). We chose two-sample t-test of equal variance for the purpose. As there were three groups, so Bonferroni correction was used to find corrected post-hoc t-test p-value. For Bonferroni correction, P-value of 0.05 was divided by number of comparison groups that yield p-value of 0.016. So, p-value of 0.016 or less was considered significant for post-hoc t-test between two groups. Pearson chi-square test was utilized to see statistical difference among groups for gender difference, difficulty in weaning from CPB and mortality. Chi-square test was used to perform comparison between groups for categorical variables like weaning difficulty from CPB, mortality and moderate PR between Group-II and III. PCS test was not performed for moderate PR as Group-I has inherent free PR related to operative procedure

## RESULTS

Table-I summarized the baseline characters of patient population, operative information and early operative outcome that underwent surgical repair of TOF with TAP (Group-I) or MC repair (Group-II) or PV repair (Group-III). There was statistically significant difference regarding age of patients in all three group however post-hoc t-test showed differences was between Group-I & II, Group-I & Group-III but no difference of age when Group-II was compared to Group-III with p-value of 0.17. There was no statistical difference regarding gender distribution in all three groups with p-value of 0.43 by Pearson chi-square test. ANOVA test showed p-value of <0.54 (non-significant) for weight comparison of all three groups and similar trends were observed for post-hoc t-test as well as shown in Table-II. Operative finding showed that the total aortic cross clamp time and total cardiopulmonary bypass time (CPB) was significantly different for all three groups with p-value <0.0001. These were higher for Group-II as compared to Group-III and I as construction of mono-cusp is technically challenging and time consuming. Post-hoc t-test also showed significant p-value for aortic cross clamp time and CPB time when groups were compared with each other. The incidence of difficult wean from CPB was statistically different among all three group with p-value of 0.006. However, when group compared with each other it revealed that Group-I have statistically significant incidence of difficult weaning of CPB from Group-II and III with p-values of 0.016 and 0.0007 respectively. However, there was no difference in weaning of CPB in-Group-II and Group-III with p-value of 1. Post-operative mechanical ventilation was also different in all three groups with p-value of <0.0001 and it show longer duration for Group-I. Post-hoc t-test however reveal that that there was no significant difference between MC repair and PV groups. Post-operative ICU stay showed longest stay in Group-I with p-value of <0.0001 when compared to Group-II and Group-III. However post-hoc t-Test revealed that there was no difference when Group-I was compared to II. In addition, post-operative ICU stays was longer in-Group-II than Group-III with p-value of < 0.0001. Hospital stay duration was shorter in-Group-III as compared to Group-I and II, with p-value of <0.0001. Post-hoc t-test showed there were no statistical difference between groups I and II regarding hospital stay with p-value of 0.12. The incidence of operative mortality was not different among groups with p-value of 0.15. Difference of operative mortality was not statistically significant when comparison was done between groups as shown in Table-II. A separate group comparison by chi-square test between Group-II and Group-III showed statistically significant incidence of moderate PR measured by pre-discharge echocardiography in-Group-II with p-value of 0.0052.

**Table-I:** Baseline characteristic, operative information and post-operative outcome of all three groups of TOF surgery. Transa-annular Patch repair (TAP), Mono-cusp Repair (MCR), Pulmonary valve repair (PVR), Analysis of Variance (ANOVA), Pearson Chi-square test (PCS).

Clinical parameter	Group-I (TAP)	Group-II (MCR)	Group-III (PVR)	P-value
Age (years)	12.3 ± 6.34	16.09 ± 5.78	13.45 ± 7.76	< 0.0001 (ANOVA)
*Gender*				
Male	37.5%	36.17%	46.88%	0.43 (PCS)
Female	62.5%	63.82%	53.22%	
Weight (kg)	33.20 ± 10.9	35.55 ± 9	33.95 ± 12.43	0.54 (ANOVA)
Aortic cross clamp time (mins)	76.83 ± 13.82	113.38 ± 14.86	64.95 ± 6.9	< 0.0001 (ANOVA)
CPB Time (mins)	115.5 ± 19.70	153.68 ± 17.82	98.70 ± 11.71	< 0.0001 (ANOVA)
Ventilation time (Hrs)	9.32 ± 3.60	8.9 ± 1.84	6.53 ± 1.4	< 0.0001 (ANOVA)
Difficult Weaning from CPB	23.44%	4.25%	3.125%	0.0006 (PCS)
ICU stay (hrs)	31.42 ± 11.18	29.57 ± 9.4	21.18 ± 5.26	< 0.0001 (ANOVA)
Hospital Stay (days)	8.33 ± 2.60	8.49 ± 1.25	5.92 ± 0.72	< 0.0001 (ANOVA)
Operative Mortality	7.81%	2.13%	1.56%	0.15 (PCS)

**Table-II:** Post-hoc t-test and Boneferroni Correction and chi-square test for categorical variables for Baseline characteristic, operative information and post-operative outcome of all three groups of TOF surgery. Transa-annular Patch repair (TAP), Mono-cusp Repair (MCR), Pulmonary valve repair (PVR).

Clinical parameter	Group-I vs Group-II	Group-I vs Group-III (p value)	Group-II v Group-III (p value)
Age	< 0.0001	0.17	< 0.00023
Weight	0.1	0.8	0.17
Aortic cross clamp time (mins)	< 0.0001	0.04	< 0.0001
CPB Time (mins)	< 0.0001	< 0.0001	< 0.0001
Difficult Weaning from CPB	0.016	0.007	1
Ventilation time (mins)	< 0.0001	0.04	< 0.0001
ICU stay (days)	0.07	< 0.0001	< 0.0001
Hospital stay (days)	0.12	< 0.0001	< 0.0001
Operative mortality	0.18	0.94	0.82

**Table-III:** Incidence of moderate pulmonary regurgitation on echocardiography done before hospital discharge.

Clinical Parameter	Group-II	Group-III	P-value
Moderate PR	19.1%	3.125%	0.0052

These results showed that MC repair do have some beneficial impact in TOF surgery when compared to TAP group with regards to early postoperative outcome i.e. Weaning from CPB, post-operative mechanical ventilation duration, ICU-stay, Hospital stay duration and operative mortality. However, this benefit is limit to ease in weaning from CPB and postoperative mechanical ventilation only when measure statistically by p-value. PV repair showed most impressive impact over early outcome when compared to TAP and MC repair.

## DISCUSSION

TAP and PV repair are two extreme strategies of RVOTO repair utilized for TOF surgery. Severe PV annular hypoplasia makes TAP as an inevitable choice particularly when Z score is very low. Higher Z score for PV annulus and good PV leaflets anatomy favors PV sparing for TOF surgery. Some surgical groups even try to preserve PV function even in borderline Z score of PV annulus and accept residual gradient across RVOT (up to 20-30 mmHg).^9^ The scientific reason behind this approach is saving the RV from immediate free pulmonary regurgitation of TAP and it sequela. This free inherent PR of TAP in presence of peri-operative hypoxemia, myocardial resection, ischemic re-perfusion injury echoes as higher incidence of low cardiac out-put state (LCOS), prolong post-operative mechanical ventilation, longer ICU & hospital stay and mortality.^10^,^11^ Long term impact of free PR is RV dysfunction, higher incidence of redo-surgery and poor prognosis.^12^ These facts collectively pave the road to employ operative strategies to add competence en rout to PA during TOF repair. Various surgical techniques have been described for the purpose. Mono-cusp repair is one of these. Pericardium, PTFE, Contegra (valve conduit) etc. are utilized to create mono-cusp within TAP that halts the free PR of isolated TAP repair. This saves hypertrophied RV to face deleterious free PR of sudden onset.

Sasson L et al. had demonstrated that mono-cusp repair showed better clinical outcome in the form of shorter postoperative mechanical ventilation, short ICU & hospital stay in TOF repair when compared to TAP only with no impact over operative mortality.^7^ Their study also reports beneficial impact of mono-cusp over RV hemodynamics including RV pressures, LV/RV pressure ratio that explains the better clinical outcomes. Similar outcome is reported by He GW et al., Anagnostopoulos P et al. and Sasikumar D et al.^13^-^15^ Study by Ismail SR et showed that mono-cusp repair with Contegra in their TOF population showed better profiles of postoperative ventilation, ICU stay, Hospital stay and mortality when compared to TAP only but however this benefit did not appear reach statistical significance.^16^ Singh NM et al also reported lack of advantage of MC repair over early outcomes of TOF repair.^17^ Our study population demonstrated that MC repair help to liberate from CPB without difficulty and patient population has to face few hours of post-operative mechanical ventilation. However, impact of MC repair over ICU stay, Hospital stay and operative mortality is less profound. Wankhade PR et al. also reports finding similar to our study.^18^

Literature has shown a clear advantage of PV repair strategy of RVOT repair in TOF with much fewer postoperative hours of mechanical ventilation, shorter ICU & hospital stay and operative mortality.^19^ Technically it saves lot of operative time with short aortic cross clamp time and CPB time and hence betters early operative outcomes. Long-term outcome of PV sparing is also promising.^20^,^21^ Likewise, TAP had shown promising results over period of decades but when compared to PV sparing it showed less than optimal results from short-term and long term outcome point of view.^21^ Although MC repair is showing better outcomes profile when compared to TAP, but yet it has to establish its definitive role from prognostic stand point. MC repair showed more incidence of moderate PR when compared to PVR in our study population. What we believe that more and more experience, expertise and supportive data can make outlook of MC repair strategy as prominent option among other strategies to deal RVOT of TOF. At the same time, long term durability of MC repair strategy is also debatable so more data is required to know about benefits of MC repair on long term out of TOF repair.^22^

## CONCLUSION

PV repair strategy should be employed for RVOT repair of TOF whenever feasible. MC repair showed fewer hours of postoperative mechanical ventilation and higher incidence of easy weaning from CPB when compared to TAP, however its impact over ICU stay, Hospital stay and operative mortality is not profound in our TOF repair population.

### Author’s Contribution:

**TW:** Conceived, designed the research methodology, prepared this manuscript and is accountable for the originality of the research work.

**MZAA and KK:** Did data analysis, helped in writing the manuscript and reviewed the manuscript.
